# Evaluation of the accuracy of an enhanced MLC leaf model for Linac‐based stereotactic radiosurgery

**DOI:** 10.1002/acm2.70143

**Published:** 2025-07-13

**Authors:** Yun Yang, Khayrullo Shoniyozov, Taoran Li, Virginia Lockamy, Michael Bieda, Boon‐Keng Kevin Teo, Suneel Nagda, Michelle Alonso‐Basanta, Wenbo Gu

**Affiliations:** ^1^ Department of Radiation Oncology University of Pennsylvania Philadelphia Pennsylvania USA; ^2^ Varian a Siemens Healthineers Company Palo Alto California USA

**Keywords:** DLG, ELM, Linac‐based SRS, MLC

## Abstract

**Purpose:**

For LINAC‐based stereotactic radiosurgery (SRS) treatments, the binary MLC models utilizing single dosimetric leaf gap (DLG) parameters in Eclipse versions prior to v18 can result in imperfect agreement between measured and calculated doses, increased commissioning complexity, and user‐dependent variability. This study aims to evaluate the efficiency and accuracy of the enhanced leaf model (ELM) in Eclipse version 18.0, which incorporates the actual rounded‐end MLC design in dose calculations.

**Methods:**

ELM parameters were determined from measurements and configuration in a test Eclipse v18.0 system for an Edge LINAC with High Definition MLC (HDMLC) and a TrueBeam LINAC with the Millennium 120‐leaf MLC (M120). The anisotropic analytical algorithm (AAA) was used to calculate doses for both 10FFF and 6FFF energies. The v18 ELM model was compared to the current version 16.1 (v16) model, which utilized single DLG parameter. Dose calculations were performed and compared for (1) static small on‐axis fields, (2) static small off‐axis fields, (3) single‐isocenter single‐target (SIST) HyperArc plans, and (4) single‐isocenter multiple‐target (SIMT) HyperArc plans. Gafchromic EBT4 film and myQA SRS device were used for dose verification.

**Results:**

The measurement required for ELM was similar to that of the original DLG, but ELM configuration provided significant time savings. The measurements showed comparable or improved accuracy with the ELM model for both static fields and patient‐specific plans. A significant improvement in dose calculation accuracy was observed with the ELM particularly for SIMT patients with a large number of small targets.

**Conclusion:**

The new ELM introduced in Eclipse v18 substantially improves efficiency and consistency of the modeling process in the Eclipse dose calculation algorithm while maintaining comparable or superior accuracy for Linac‐based SRS.

## 1 INTRODUCTION

Stereotactic radiosurgery (SRS) has become a standard treatment for patients with 1–10 brain metastases due to its ability to achieve local tumor control while minimizing neurocognitive decline compared to whole‐brain radiotherapy (WBRT).^1^, ^2^ The adoption of linear accelerator (LINAC)‐based arc therapy in SRS has increased in recent years, driven by its broad accessibility, frameless immobilization, and technological advancements that allow high conformality and efficiency. LINAC‐based SRS enables adaptive planning and shorter treatment times, making it suitable for both single‐ and multi‐target intracranial treatments.

Precise delivery of conformal radiation doses in SRS requires accurate modeling of the multileaf collimator (MLC), which shapes the beam aperture dynamically. A common design of MLCs features single‐focused, tongue‐and‐groove leaves with rounded ends, as seen in systems such as Varian Millennium 120‐leaf MLC^3^, Varian High Definition MLC,^4^ and Elekta Agility MLC.^5^ While the rounded leaf tip helps achieve a relatively uniform penumbra across the field, it also introduces complexities in dose modeling, particularly in small and off‐axis fields. Small deviations in MLC modeling can lead to notable discrepancies in calculated dose, especially in highly modulated, small field conditions typical of SRS.^6^, ^7^, ^8^, ^9^, ^10^

In Eclipse treatment planning system (TPS) versions up to v17 (Varian Medical Systems, Palo Alto, California, USA), the MLC is modeled using a simplified binary‐state approach. In this model, the leaves are modeled as a rectangular block with a flat end and is assigned a user‐defined transmission factor (Tr).^11^ A second key parameter, the dosimetric leaf gap (DLG), accounts for both the physical gap between leaves and the penumbra shift caused by the rounded leaf tips. Although DLG can be measured, the values that best match clinical dose measurements often differ from those obtained in initial testing.^10^ As a result, DLG typically requires manual optimization using representative clinical plans to achieve accurate dose calculation. This process is especially important—and more challenging—for SRS, where small field sizes and tight dose gradients demand higher modeling precision.^12^ In single‐isocenter multiple‐target (SIMT) SRS plans, the use of a single DLG value becomes even more limiting, as it cannot accurately capture both on‐ and off‐axis leaf behaviors. As a result, iterative tuning of DLG is often needed during commissioning, adding significant complexity and time to the process.^13^

To address these limitations, Eclipse version 18 introduced an Enhanced Leaf Model (ELM) that incorporates the actual rounded leaf‐end geometry into dose calculations. This model eliminates the need for DLG tuning and instead uses a ray‐tracing approach to account for MLC geometry, including tip curvature and body thickness. Studies have shown that the ELM improves dose agreement for sliding gap fields,^14^, ^15^ but its performance in clinically relevant SRS scenarios has not yet been fully evaluated.

In this study, we assess the dosimetric accuracy and commissioning efficiency of the ELM in the context of LINAC‐based SRS. Comparisons are made against the traditional DLG‐based model in Eclipse v16 using small field measurements and patient‐specific HyperArc SRS plans. Evaluations are performed across two MLC designs, multiple photon energies, and using both film and high‐resolution detector array measurements. Our goal is to determine whether the ELM improves modeling accuracy for complex SRS cases and to provide practical guidance for clinical implementation.

## 2 METHODS

### 2.1 ELM configuration

The evaluation of the ELM was performed on two Varian LINACs: a TrueBeam Edge machine equipped with High Definition 120 MLC (HDMLC) and a TrueBeam machine with Millennium 120 MLC (M‐120 MLC). Both MLC models consist of 60 leaf pairs. For the HDMLC, the innermost 32 pairs of tungsten leaves are 2.5 mm wide, while the 28 outer pairs are 5.0 mm wide, as projected to the isocenter. The Millennium 120 MLC has 40 inner leaf pairs that are 5.0 mm wide and 20 outer pairs that are 10.0 mm wide at the isocenter. The Millennium 120 has a leaf‐end radius of curvature of 8 cm, compared to 16 cm for the HDMLC.

Both machines used the anisotropic analytical algorithm (AAA) modeled in Eclipse v16.1 for clinical treatments. For the Edge machine, the 10FFF (flattening filter‐free) beam model was commissioned for SRS treatments, with DLG tuned to optimize the patient specific quality assurance (PSQA) passing rate for SIMT cases. The 6FFF energy was not commissioned for SRS and the DLG was instead tuned for 3D/IMRT/VMAT treatments. For the TrueBeam, both 6FFF and 10FFF were configured for 3D/IMRT/VMAT treatments.

ELM parameters were measured for each machine and energy using a PTW N30004 Farmer‐type ion chamber and solid water phantoms. Measurements were taken at 10 cm depth with a 100 cm source‐axis distance (SAD).^16^ The fields included an open field, two transmission fields (one for each MLC bank), and only three sweeping gap fields (4 , 6 , and 20 mm) compared to seven sweeping gap fields (2–20 mm) for the DLG measurement.^17^ It is worth noting that these measurements were performed for the purpose of beam modeling and MLC configuration, not for direct small‐field dosimetry. In Eclipse TPS, the same MLC model is applied uniformly across both small and conventional field sizes.

For Eclipse version prior to v18, transmission factors and DLG were calculated and input into RT Administration. For Eclipse v18, ion chamber readings were directly entered into Eclipse under Beam Configuration to derive the ELM parameters. In this study, ELM was configured for AAA algorithm in an Eclipse v18 test environment. All non‐MLC beam data were transferred from the v16 clinical system to ensure consistency between v16 and v18. The effective target spot sizes in both the X and Y directions were set to 0 mm for the AAA algorithm with MLC‐in‐field calculations in both v16 and v18, following the recommendations in the Eclipse algorithm refence.^11^ While fine‐tuning the effective source size could potentially further improve dose calculation accuracy, particularly in penumbra modeling, this optimization was beyond the scope of the current study.

Unlike previous Eclipse versions where the MLC was modeled as a uniform block with a flat end, the ELM introduced in v18 incorporates the detailed physical geometry of each MLC type. This includes the rounded leaf tip, the drive screw cutout, and variations in leaf body thickness for both the HDMLC and Millennium 120 MLC. These geometric characteristics are predefined in the system and used during beam modeling. In the configuration process, sweeping gap measurements are directly entered into the beam configuration module, where the system applies a ray‐tracing algorithm to simulate attenuation through the realistic MLC geometry. A curve‐fitting procedure is then used to determine the optimal transmission and leaf gap parameters that minimize discrepancies between calculated and measured sweeping gap doses. Although the leaf gap parameter is still referred to as the “dosimetric leaf gap,” it no longer represents a physical gap measurement; rather, it functions as a fitting parameter that adapts the predefined geometric model to empirical data.

### 2.2 Verification measurements

Small‐field and patient‐specific plans were used to evaluate the accuracy of the ELM and to compare it with the previous model. For the HDMLC, the small fields included six on‐axis MLC‐shaped fields and one off‐axis MLC field. The on‐axis fields consisted of 4×4 cm^2^, 3×3 cm^2^, 2×2 cm^2^, 1×1 cm^2^, 0.5×0.5 cm^2^ square fields, and a 1 cm^2^ circular field. The off‐axis fields had an opening of 1×1 cm^2^, extending up to 5 cm from the isocenter in the x‐direction (leaf travel direction), and a 1×1 cm^2^ opening extending up to 6 cm from the isocenter in the y‐direction (perpendicular to leaf travel). For the Millennium 120, the small fields included four on‐axis MLC fields—4×4 cm^2^, 3×3 cm^2^, 2×2 cm^2^, and 1×1 cm^2^—and one off‐axis field with a 1×1 cm^2^ opening. All small fields were created for both 6FFF and 10FFF energies.

Six single‐isocenter single‐target (SIST) and three single‐isocenter multiple‐target (SIMT) intracranial SRS patients were retrospectively selected for patient‐specific evaluation. The six SIST patients had a range of PTV volumes, from 0.09 to 2.4 cc (S‐Pt‐01 to S‐Pt‐06), representing a wide variety of volumes to assess the impact of the ELM. The SIMT patients were chosen to provide a diverse range in the number of PTVs, their volumes, and off‐axis distances for each PTV (M‐Pt‐01 to M‐Pt‐03). Patient details are summarized in Table 1. For both the HDMLC and Millennium 120 MLCs, 6FFF and 10FFF plans were created using the HyperArc technique. The dose calculation grid size was 1 mm.

**TABLE 1:** Characteristics of the investigated six SIST and three SIMT patient cases.

Patient	Number of PTVs	Range of PTV volumes (cc)	Range of PTV center to isocenter distance (cm)	Prescribed dose and fractionation
S‐Pt‐01	1	0.3	−	21 Gy /1fx
S‐Pt‐02	1	0.09	−	21 Gy/1fx
S‐Pt‐03	1	0.77	−	21 Gy/1fx
S‐Pt‐04	1	1	−	21 Gy/1fx
S‐Pt‐05	1	1.9	−	21 Gy/1fx
S‐Pt‐06	1	2.4	−	21 Gy/1fx
M‐Pt‐01	8	0.17–2.64	2.90–9.49	3 lesions to 20 Gy/1fx, 5 lesions to 21 Gy/1fx
M‐Pt‐02	4	0.39–4.06	1.78–5.24	30 Gy/5fx (All)
M‐Pt‐03	2	1.9–2.4	4.54	21 Gy/1fx (All)

For both small fields and patient‐specific plans, the original dose distribution was created using the Eclipse v16 model and then recalculated with the v18 model incorporating the ELM. Gafchromic EBT4 films (Ashland Inc., Bridgewater, New Jersey, USA) were used to measure and compare the accuracy of the ELM model. The films were placed horizontally at a 10 cm depth within a solid water phantom, with an additional 10 cm of solid water underneath to ensure adequate backscatter.

For small fields and SIST patients, the source‐to‐axis distance (SAD) was set to 100 cm, with the films positioned at the center of the field. In SIMT cases, the solid water and film were shifted to align with the center of a selected PTV, allowing the films to capture off‐axis high‐dose regions. To eliminate the influence of couch mechanical inaccuracies, no couch kicks were applied during patient‐specific plan measurements. The manufacturer‐specified dose range for EBT4 films is 0.2–10 Gy, which is below the prescribed dose levels for patient plans, as outlined in Table 1. Therefore, the patient plans were renormalized to adjust the maximum doses to fall between 5 and 10 Gy. The was corrected by reference ion chamber (Exradin A12) and setup cross calibrated during annual QA. A reference ion chamber (Exradin A12) was used to verify the machine output variation of a 10×10 cm^2^ open field, on the day of film irradiation. The films were scanned at 300 dpi resolution.

Gafchromic EBT4 films were calibrated using a set of known dose exposures ranging from 0.2  to 10 Gy. Films were irradiated in a solid water phantom with a 10×10 cm^2^ field at 5 cm depth and 100 cm SAD using both 6FFF and 10FFF beams. Each calibration level was delivered in triplicate to enhance statistical robustness. Delivered doses were verified using the Exradin A12 chamber.

After a 24‐h post‐irradiation stabilization period, films were scanned with an Epson Expression 10000XL flatbed scanner in transmission mode, using consistent orientation and acquisition settings. To correct for scanner lateral response non‐uniformity, a uniformly irradiated 2 Gy film was scanned at multiple lateral positions. Pixel intensity variations were used to derive a lateral correction factor, which was applied to all small fields and patient‐specific QA film analyses.

In addition to EBT4 films, myQA SRS detector array (IBA Dosimetry, Schwarzenbruck, Germany) was utilized as a secondary verification method. This solid‐state device features a 12 × 14 cm^2^ CMOS matrix with a 0.4 mm resolution.^18^ For small field and SIST plans, the detector plane was positioned horizontally at isocenter, parallel to the coronal plane. For SIMT cases, the detector plane was rotated about its longitudinal axis to optimize coverage and capture dose distributions across multiple off‐axis targets.

All measurements were compared with the original v16 model and the new v18 ELM model. To account for setup uncertainties, a rigid transformation was applied to the measured dose profiles prior to gamma analysis to achieve optimal alignment with the calculated dose distributions. Gamma passing rates were calculated using a 3% DD (dose difference) /1 mm DTA (distance to agreement), and 1%/1 mm criteria with global normalization and a 10% threshold, employing an in‐house developed MATLAB script. Additionally, a more stringent 0.5%/0.5 mm gamma passing rate was also evaluated. Due to the inherent limitations of film dosimetry and solid‐state detectors in absolute dose accuracy, the 0.5%/0.5 mm gamma passing rate was used primarily as a relative comparison metric to evaluate the agreement of dose profiles under stringent criteria. While this fine‐resolution metric provides insight into submillimeter dose modeling differences, it is subject to increased measurement uncertainty.

### 2.3 Matched machine comparison

In institutions with multiple similar LINACs, a common practice is to dosimetrically match the machines. When LINACs exhibit the same or nearly identical dosimetric characteristics, patients can be moved between machines without altering their treatment plans, allowing for greater scheduling flexibility and increased efficiency during machine downtimes. For dosimetrically equivalent machines, an identical beam model is typically used in the TPS. In earlier versions of Eclipse (prior to v18), transmission factors and DLG values were derived and fine‐tuned and thus one set of Tr and DLG parameters can accommodate and be used across all machines.

ELM in Eclipse v18 is designed to simplify and standardize transmission factor (Tr) and DLG configuration with minimum measurements required for each machine. After configuration, it is possible to manually edit and fine tune Tr and DLG values. However, modifying the parameters will remove measurement and comparison data and raise questions for dosimetrically equivalent machines. These include how the beam model differs when measurements are taken from each machine and which parameters should be used for shared beam data. This issue is particularly relevant during the Eclipse upgrade process, especially for SRS treatments where dose calculations are more sensitive to beam model accuracy.

To address these questions, we measured ELM parameters on a second TrueBeam LINAC equipped with Millennium 120 MLC (TB2), which was dosimetrically equivalent to the first TrueBeam (TB1) described in Section II.1. In the v16 beam models, both TB1 and TB2 shared identical transmission factors and DLG values, resulting in the same v16 algorithms. ELM was separately configured using measurements from each machine, creating two v18 models, V18‐TB1 and V18‐TB2. The film and myQA SRS measurements were performed on TB1. The resulting measurements were then compared with V18‐TB1 and V18‐TB2 to evaluate the differences in ELM configurations between the dosimetrically equivalent machines.

## 3 RESULTS

### 3.1 MLC configuration

The ion chamber readings from sweeping gap measurements were input into the Eclipse v18 environment to configure the MLC parameters. The resulting Tr and DLG are shown in Table 2, with no further adjustments made to these values. Although the leaf gap parameter in v18 serves a different purpose than the original DLG concept, the term “dosimetric leaf gap” has been retained. For comparison, the transmission factors and optimized DLGs from the original v16 model are also presented in Table 2. In the v16 model, DLG values were obtained by extrapolating sweeping gap results to zero dose, representing the mechanical leaf gap and the partial attenuation from the rounded leaf tip. Manual and empirical optimization of DLG was also performed for improved agreement with test plans such as using the dynamic chair pattern, TG119 test plans,^19^ and patient‐specific plans.

**TABLE 2:** The DLG and transmission factor (Tr) comparison between v16 and v18 for the three tested machines.

	V16		V18	
	Tr	DLG (cm)	Tr	DLG (cm)
HDMLC, 6FFF	0.0075	0.02	0.0099	−0.058
HDMLC, 10FFF	0.013	0.075	0.012	−0.051
M‐120, TB1, 6FFF	0.008	0.12	0.012	−0.015
M‐120, TB1, 10FFF	0.013	0.15	0.014	−0.0055
M‐120, TB2, 6FFF	0.008	0.12	0.012	−0.012
M‐120, TB2, 10FFF	0.013	0.15	0.014	−0.0085

In the new ELM model, the beam attenuation already accounts for the rounded leaf tip, and the DLG is fitted within the beam configuration module in Eclipse to minimize discrepancies between calculated and measured sweeping gap deliveries. As a result, the DLG values for different energies and machines in the ELM model were negative for the tested machines. For the HDMLC, the ELM v18 DLGs were ‐0.058 cm for 6FFF and ‐0.051 cm for 10FFF, compared to the v16 optimized values of 0.02  and 0.075 cm, respectively.

For the two dosimetrically equivalent TrueBeam machines, the same set of v16 transmission factors and DLG were determined for clinical use. In v18, the configured transmission factor remained the same between the two machines, with only slight differences in DLG (around 0.003 cm) observed for both energies. Theoretically, the transmission factor between v16 and v18 should remain unchanged, as the same calculation method—comparing transmission fields to open fields—is used. However, when configuring the ELM, a new set of measurements was performed rather than relying on the original v16 data, leading to a transmission factor difference of up to 0.004 between v16 and v18.

### 3.2 On‐axis small fields validation

**FIGURE 1:**
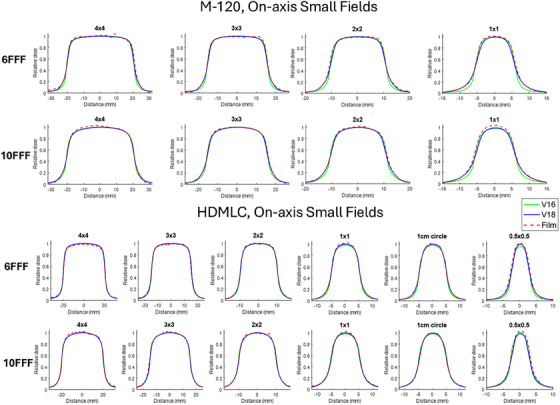
On‐axis small field profile comparison for M‐120 (top two rows) and HDMLC (bottom two rows), across v16 (green solid line), v18 (blue solid line), and film measurements (red dashed line). Dose profiles are shown for both 6FFF and 10FFF energies. M‐120 field sizes range from 4×4  to 1×1 cm^2^, while HDMLC field sizes range from 4×4  to 0.5×0.5 cm^2^. The central dose profiles are plotted along the MLC leaf travel direction.

The central dose profile comparisons between film measurements and calculations along the MLC travel direction for centered small fields are shown in Figure 1. The dose distributions from Eclipse v16, v18, and measurements were normalized to the maximum dose value from the v18 calculation. For M‐120, the v16 and v18 dose calculations differ most in the penumbra regions, with the v18 penumbra being less sharp than v16. This difference becomes more pronounced as the field size decreases to 1×1 cm^2^ and is observed for both 6FFF and 10FFF energies. The central axis doses between v16 and v18 are similar. Film measurements, however, show better agreement with the v18 ELM model in the penumbra, especially in regions below 50% dose. For 2×2 cm^2^ and 1×1 cm^2^ fields, v16 shows a slightly wider shoulder region, with film measurements agreeing better in that area. The gamma passing rates comparing calculated doses to film measurements are listed in Table 3 for M‐120. For 10FFF, the 3%/1 mm and 1%/1 mm passing rates are similar between v16 and v18 for the 3×3 cm^2^ and 4×4 cm2 fields. For the smaller 2×2 cm^2^ and 1×1 cm^2^ fields, v18 improves the 1%/1 mm passing rates by 5.1% and 13.5%, respectively, compared to the original v16 model. For 6FFF, v18 improves the gamma passing rate across all on‐axis small fields, especially for the 1×1 cm^2^ field, where the 1%/1 mm passing rate increases from 87.5% to 99.1%. Gamma passing rate comparisons with myQA SRS measurements are also listed in Table 3, and the results align with the film measurements.

**TABLE 3:** Gamma passing rate comparison between calculations and measurements for M‐120 under small fields.

		Fields size (cm^2^)	4 × 4		3 × 3		2 × 2		1 × 1		Off axis	
Energy	Device	Algorithm	V16	V18	V16	V18	V16	V18	V16	V18	V16	V18
10FFF	Film	3%/1 mm	91.9%	93.1%	100.0%	100.0%	99.8%	100.0%	90.9%	97.8%	97.7%	100.0%
		1%/1 mm	75.1%	76.5%	96.1%	97.2%	88.9%	94.0%	83.2%	96.7%	95.3%	99.4%
		0.5%/0.5 mm	56.0%	64.0%	74.6%	85.9%	63.5%	79.4%	68.8%	88.2%	91.0%	96.1%
	myQA SRS	3%/1 mm	100.0%	100.0%	100.0%	100.0%	100.0%	100.0%	99.0%	100.0%	97.8%	99.7%
		1%/1 mm	96.4%	94.9%	98.8%	98.1%	100.0%	100.0%	94.4%	99.9%	94.6%	98.5%
		0.5%/0.5 mm	81.4%	76.3%	89.4%	89.3%	87.8%	96.1%	76.2%	94.5%	89.3%	94.3%
6FFF	Film	3%/1 mm	97.2%	100.0%	94.4%	98.1%	92.5%	100.0%	90.9%	100.0%	97.6%	99.9%
		1%/1 mm	84.7%	89.4%	79.4%	88.4%	85.3%	94.2%	87.5%	99.1%	96.4%	99.7%
		0.5%/0.5 mm	59.2%	63.9%	52.3%	63.4%	67.3%	81.6%	75.3%	91.8%	94.1%	98.2%
	myQA SRS	3%/1 mm	100.0%	100.0%	100.0%	100.0%	100.0%	100.0%	98.0%	100.0%	96.5%	99.6%
		1%/1 mm	79.2%	81.6%	86.2%	88.2%	93.8%	94.8%	92.7%	100.0%	94.6%	99.2%
		0.5%/0.5 mm	56.3%	63.7%	72.5%	71.7%	81.6%	81.3%	78.3%	98.0%	91.1%	96.6%

Measurements were performed using both Gafchromic film and myQA SRS.

For the HDMLC, the central dose profiles for 6FFF and 10FFF are similar between v16 and v18 for fields larger than 1×1 cm^2^. Both models agree with film and myQA SRS measurements for these larger fields, with comparable gamma passing rates as shown in Table 4. For smaller 6FFF fields, the main differences between v16 and v18 doses occur in the low‐dose penumbra and central dose regions. The v18 calculated dose shows better agreement with film measurements, while the v16 model underestimates both the penumbra and central dose regions. For smaller 10FFF fields, the differences between v16 and v18 are less pronounced, with most deviations occurring in the shoulder region. Film measurements match better with v18 for these very small fields. The gamma passing rates for 10FFF small fields are not significantly different between v16 and v18, as the dose differences are relatively small. Under very strict 0.5%/0.5 mm criteria, v18 achieves film passing rates of 99.5% and 97.1% for the 1 cm circular field and the 0.5×0.5 cm^2^ square field, compared to 96.8% and 91.1% for the v16 doses.

**TABLE 4:** Gamma passing rate comparison between calculations and measurements for HDMLC under small fields.

		Fields size (cm^2^)	4 × 4		3 × 3		2 × 2		1 × 1		1 cm circle		0.5 × 0.5		Off axis	
Energy	Device	Algorithm	V16	V18	V16	V18	V16	V18	V16	V18	V16	V18	V16	V18	V16	V18
10FFF	Film	3%/1 mm	95.3%	95.9%	99.9%	99.8%	100.0%	100.0%	99.6%	100.0%	100.0%	100.0%	100.0%	100.0%	99.9%	99.9%
		1%/1 mm	85.6%	86.7%	98.1%	98.2%	99.8%	99.9%	98.2%	99.0%	100.0%	100.0%	98.5%	99.9%	99.8%	99.8%
		0.5%/0.5 mm	66.1%	65.8%	86.0%	86.9%	94.6%	96.6%	91.3%	92.8%	96.8%	99.5%	91.1%	97.1%	99.2%	99.4%
	myQA SRS	3%/1 mm	100.0%	100.0%	100.0%	100.0%	100.0%	100.0%	100.0%	100.0%	99.4%	100.0%	100.0%	100.0%	99.9%	100.0%
		1%/1 mm	99.3%	99.2%	99.0%	98.8%	100.0%	100.0%	97.8%	99.9%	97.8%	99.4%	100.0%	100.0%	99.5%	99.9%
		0.5%/0.5 mm	81.0%	83.0%	78.4%	83.6%	84.2%	90.0%	83.3%	91.8%	88.8%	93.1%	93.5%	95.0%	97.0%	98.3%
6FFF	Film	3%/1 mm	97.4%	96.8%	100.0%	100.0%	99.9%	100.0%	98.9%	100.0%	99.7%	100.0%	99.9%	100.0%	99.7%	99.9%
		1%/1 mm	90.0%	87.3%	98.0%	98.7%	96.5%	98.9%	96.1%	99.7%	98.5%	99.8%	99.8%	100.0%	99.2%	99.7%
		0.5%/0.5 mm	73.6%	74.0%	84.9%	89.6%	83.8%	89.7%	82.9%	95.5%	83.8%	98.2%	97.2%	99.9%	96.7%	98.3%
	myQA SRS	3%/1 mm	100.0%	100.0%	100.0%	100.0%	100.0%	100.0%	100.0%	100.0%	100.0%	100.0%	98.0%	100.0%	99.7%	99.9%
		1%/1 mm	94.0%	91.3%	88.8%	85.0%	94.4%	90.0%	99.1%	100.0%	97.8%	100.0%	94.9%	100.0%	99.4%	99.8%
		0.5%/0.5 mm	70.9%	66.7%	73.0%	68.3%	82.8%	80.3%	84.8%	98.0%	87.3%	100.0%	77.6%	93.8%	97.0%	98.6%

Measurements were performed using both Gafchromic film and myQA SRS.

### 3.3 Off‐axis small fields validation

**FIGURE 2:**
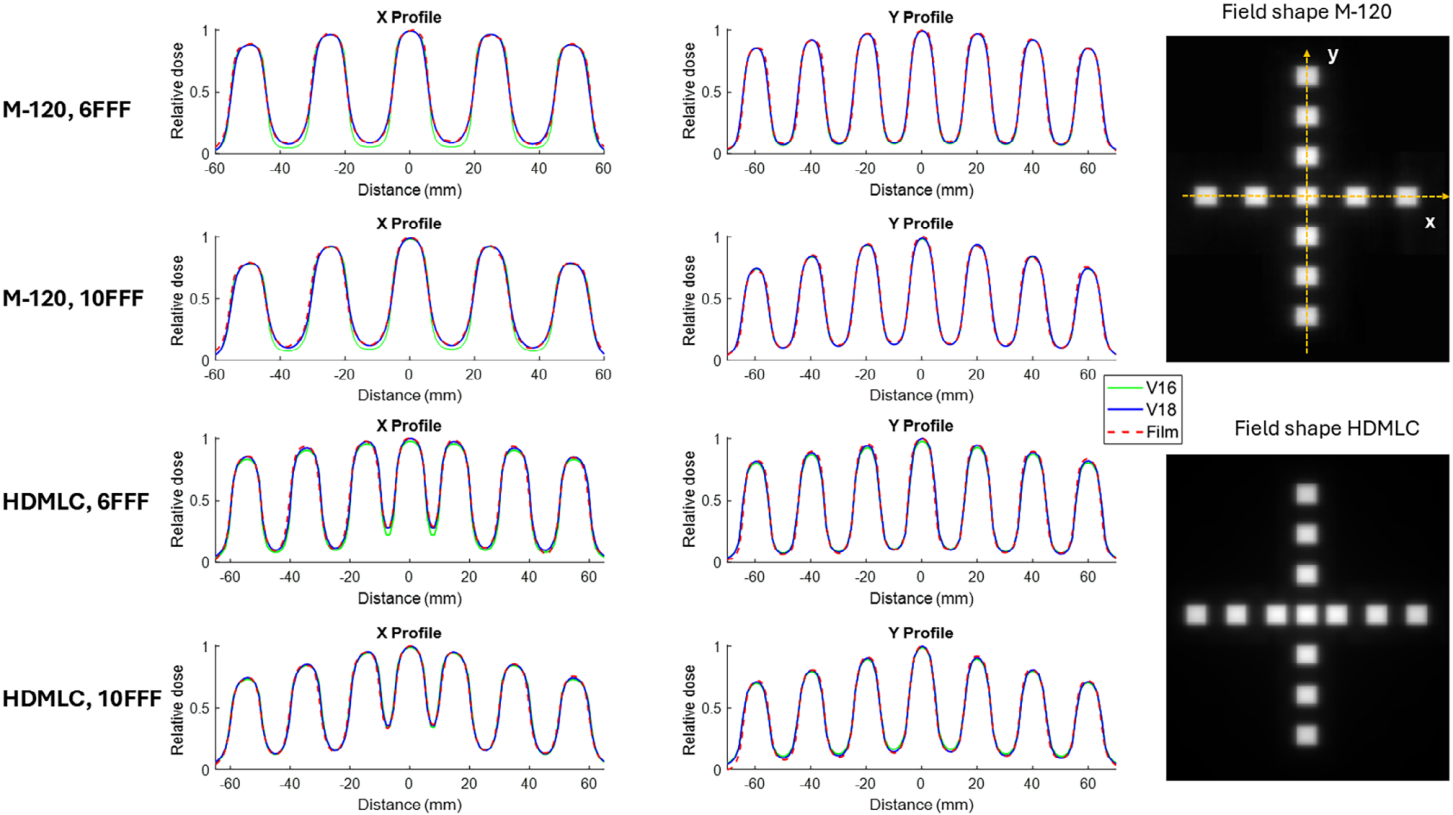
Off‐axis small fields line profile comparison between M‐120 (top two rows) and HDMLC (bottom two rows), for v16 (green solid line), v18 (blue solid line), and film measurements (red dashed line), for both 6FFF and 10FFF energies. The shape of the off‐axis field is displayed in the last column. The x‐profile and y‐profile locations are indicated by dashed yellow arrows in the field images.

The central dose profile comparisons between film measurements and calculations for off‐axis small fields are shown in Figure 2. Both the dose profiles along the MLC travel direction (x‐profile) and perpendicular to the MLC travel direction (y‐profile) are plotted.

When comparing the dose profile perpendicular to the MLC travel, the doses calculated with v16 and v18 are similar for the M‐120 with both 6FFF and 10FFF energies. For the HDMLC, however, the v18 doses are approximately 2% higher than v16 under open MLC for 6FFF and 2% lower than v16 under closed MLC for 10FFF in the y‐direction.

At different off‐axis locations along the MLC travel x‐direction, the primary difference between v18 and v16 calculated doses for the M‐120 occurs under closed MLC. Here, v16 underestimates the dose under closed leaf for both 6FFF and 10FFF, whereas v18 calculations align more closely with the film measurements. For the HDMLC, the 10FFF doses are similar between v16 and v18, but for 6FFF, the v16 doses are lower than both the v18 doses and the measured dose.

Overall, better agreement between the film measurements and v18 dose calculations is observed compared to v16. The gamma passing rates for both film and myQA SRS measurements are listed in Tables 3 and 4, with v18 showing slight improvements in passing rates over v16.

### 3.4 SIST patient‐specific validation

**FIGURE 3:**
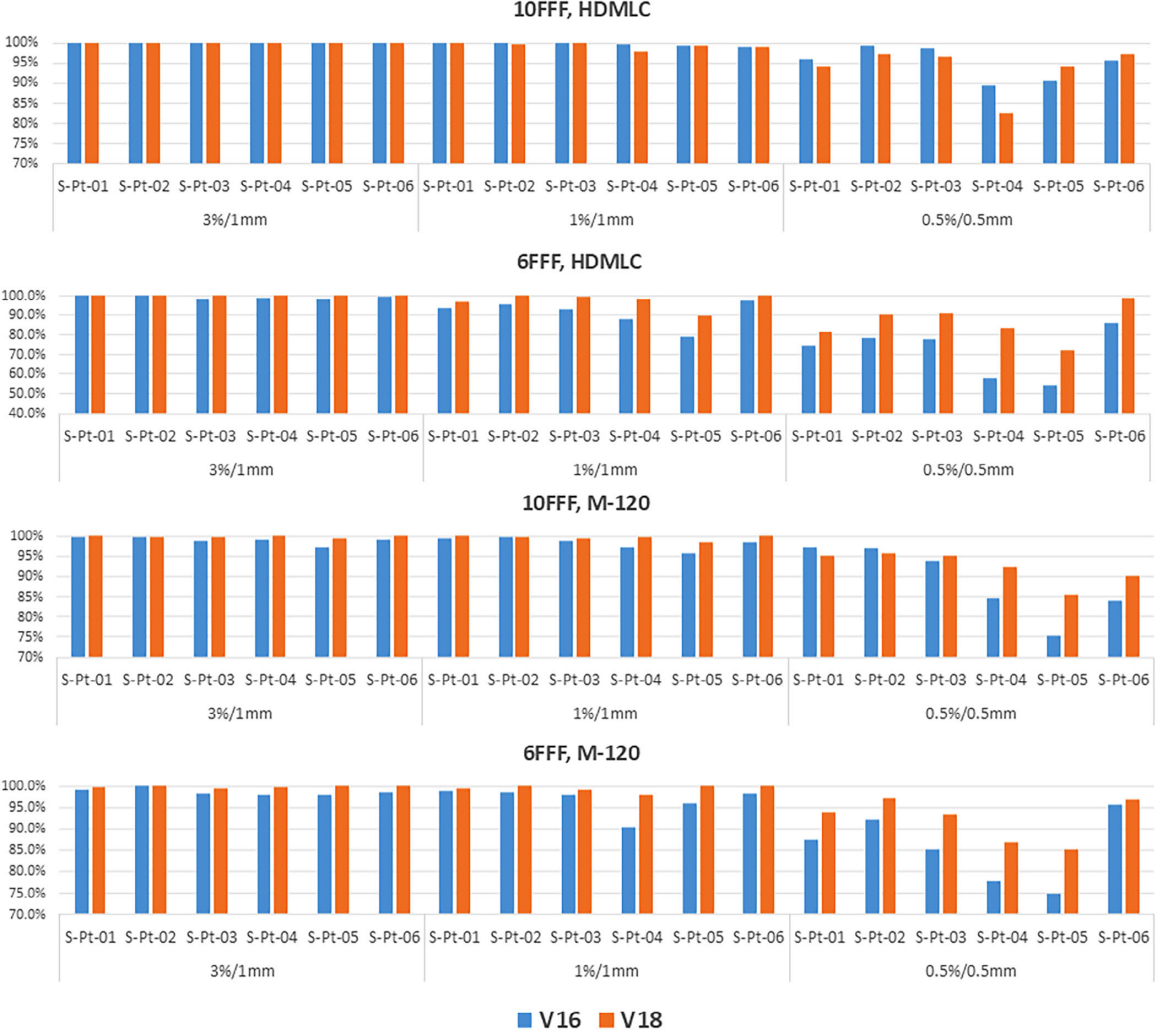
Gamma passing rate comparison at 3%/1 mm, 1%/1 mm and 0.5%/0.5 mm between film measurements and calculated doses for the six SIST patients. V16 results are shown in blue, while v18 results are represented in orange.

**FIGURE 4:**
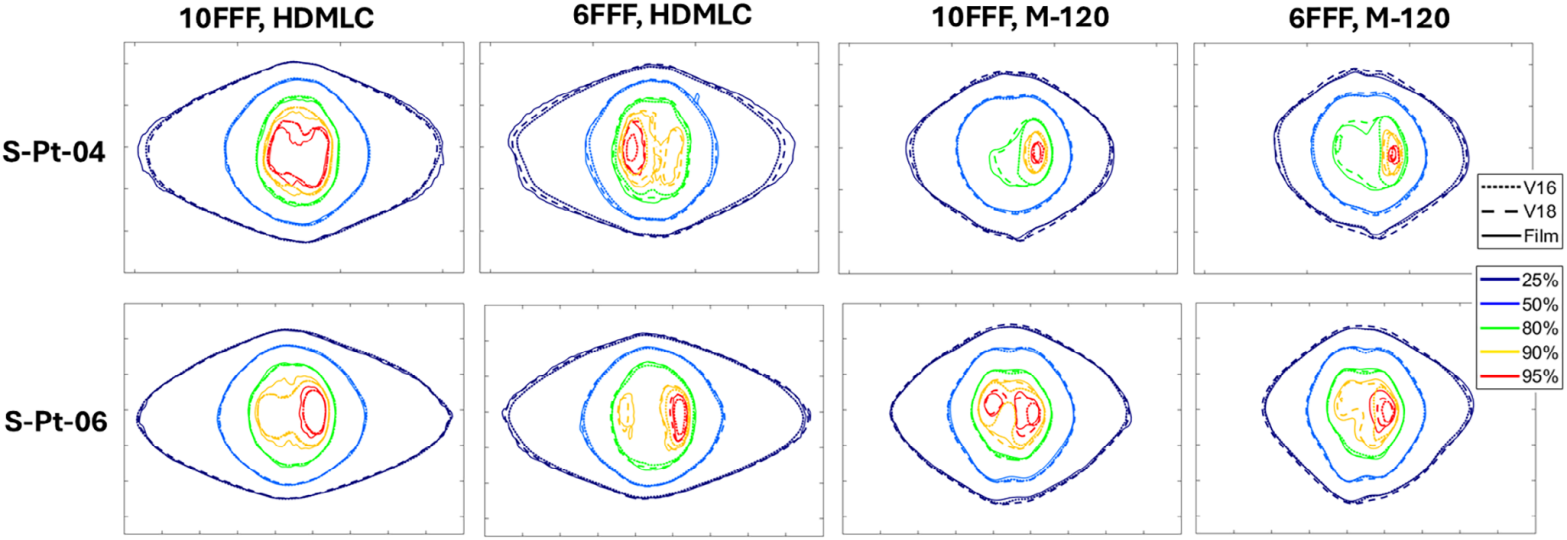
Isodose line comparison between film (solid), v16 (dotted) and v18 (dashed) at 25%, 50%, 80%, 90%, and 95% dose levels. All doses are normalized to the maximum dose of v16. The first row represents patient S‐Pt‐04, while the second row represents patient S‐Pt‐06.

The patient‐specific SIST plans were calculated using both the v16 and v18 algorithms and compared with film measurements. Gamma passing rates of 3%/1 mm, 1%/1 mm, and 0.5%/0.5 mm for the six SIST patients are shown in Figure 3, while isodose line comparisons for two patients are presented in Figure 4.

For HDMLC with 10FFF energy, v16 and v18 perform similarly across SIST patient plans, with all six plans achieving a 100% passing rate at 3%/1 mm. At 1%/1 mm, the average passing rate for v18 is 0.4% lower than for v16, with five out of six plans showing a passing rate difference of less than 0.2% between the two algorithms. At 0.5%/0.5 mm, v16 outperforms v18 in four cases, with an average improvement of 1.4%, primarily due to the slightly better agreement in low dose region with v16, as shown of 25% isodose line in Figure 4 (S‐Pt‐04). The isodose maps in Figure 4 show that v16 and v18 doses are comparable in both high‐dose and low‐dose regions.

For HDMLC with 6FFF energy, v18 shows better agreement with film measurements in all six cases. The average improvement in passing rates for 3%/1 mm, 1%/1 mm, and 0.5%/0.5 mm is 0.8%, 6.4%, and 14.8%, respectively, when compared to v16. The isodose line distributions demonstrate that the v18 ELM model improves dose accuracy in both low‐dose and high‐dose regions, with the most significant improvement seen in the dose regions above 80%. V16 underestimates high doses, while v18 aligns closely with film measurements.

For M‐120 with 10FFF, v18 outperforms v16 in four out of six cases, while the other two cases show similar results between the two algorithms. The average improvement in passing rates for 3%/1 mm, 1%/1 mm, and 0.5%/0.5 mm with v18 over v16 is 0.8%, 1.3%, and 3.7%, respectively. Dose distribution analysis in Figure 4 shows that v18 significantly improves the 80%–90% dose regions for patient S‐Pt‐04 and the 90%–95% regions for patient S‐Pt‐06, where v16 underestimates the doses.

For M‐120 with 6FFF, all six cases show similar or improved gamma passing rates and isodose agreement with v18 compared to v16. The average improvement in passing rates from v16 to v18 is 1.1%, 2.8%, and 6.8% for 3%/1 mm, 1%/1 mm, and 0.5%/0.5 mm, respectively. High‐dose regions, particularly the 80% and 90% isodose regions, are significantly improved with v18 in the test patients.

The same patient sets were also measured using the myQA SRS system, and the gamma passing rate comparison between v16 and v18 is summarized in Table 5. For HDMLC, the differences between v16 and v18 calculated doses are minimal. For M‐120, patient S‐Pt‐02, which has a small PTV of 0.09 cc, exhibits a higher gamma passing rate with v16, while the other four cases show similar results between v16 and v18.

**TABLE 5:** Comparison of gamma passing rates between calculations and myQA SRS measurements for patient‐specific plans.

		HDMLC				M120			
		10FFF		6FFF		10FFF		6FFF	
Patient number	Algorithm	3%/1 mm	1%/1 mm	3%/1 mm	1%/1 mm	3%/1 mm	1%/1 mm	3%/1 mm	1%/1 mm
S‐Pt‐01	V16	98.8%	98.7%	98.9%	98.5%	100.0%	99.3%	99.9%	91.4%
	V18	98.9%	98.8%	100.0%	99.9%	99.6%	96.7%	98.1%	91.1%
S‐Pt‐02	V16	99.7%	99.7%	99.8%	99.8%	100.0%	87.4%	98.6%	74.5%
	V18	99.7%	99.7%	100.0%	100.0%	100.0%	81.7%	89.3%	69.5%
S‐Pt‐03	V16	99.2%	99.0%	99.8%	98.3%	99.7%	96.6%	99.4%	98.4%
	V18	99.8%	99.4%	100.0%	100.0%	99.5%	94.3%	98.8%	91.9%
S‐Pt‐04	V16	98.5%	97.9%	98.5%	97.2%	99.2%	98.9%	98.6%	98.3%
	V18	98.3%	97.8%	100.0%	100.0%	100.0%	99.9%	99.9%	99.4%
S‐Pt‐05	V16	100.0%	100.0%	100.0%	98.9%	99.7%	98.1%	99.9%	98.6%
	V18	100.0%	100.0%	100.0%	99.5%	100.0%	100.0%	100.0%	98.1%
S‐Pt‐06	V16	100.0%	100.0%	100.0%	99.6%	99.0%	98.5%	100.0%	99.3%
	V18	100.0%	100.0%	100.0%	99.1%	100.0%	99.7%	100.0%	98.4%
M‐Pt‐01	V16	96.8%	92.2%	80.0%	38.8%	95.3%	63.3%	67.0%	38.2%
	V18	98.6%	97.8%	98.6%	92.3%	98.6%	87.1%	98.9%	90.1%
M‐Pt‐02	V16	96.1%	90.9%	99.8%	96.2%	98.1%	85.5%	95.3%	77.4%
	V18	98.9%	97.0%	99.0%	92.8%	98.4%	87.6%	98.1%	90.2%
M‐Pt‐03	V16	96.2%	91.7%	97.4%	79.9%	96.0%	90.2%	95.9%	83.6%
	V18	97.4%	95.8%	98.1%	92.7%	95.6%	87.9%	96.7%	90.0%

### 3.5 SIMT patient‐specific validation

**FIGURE 5:**
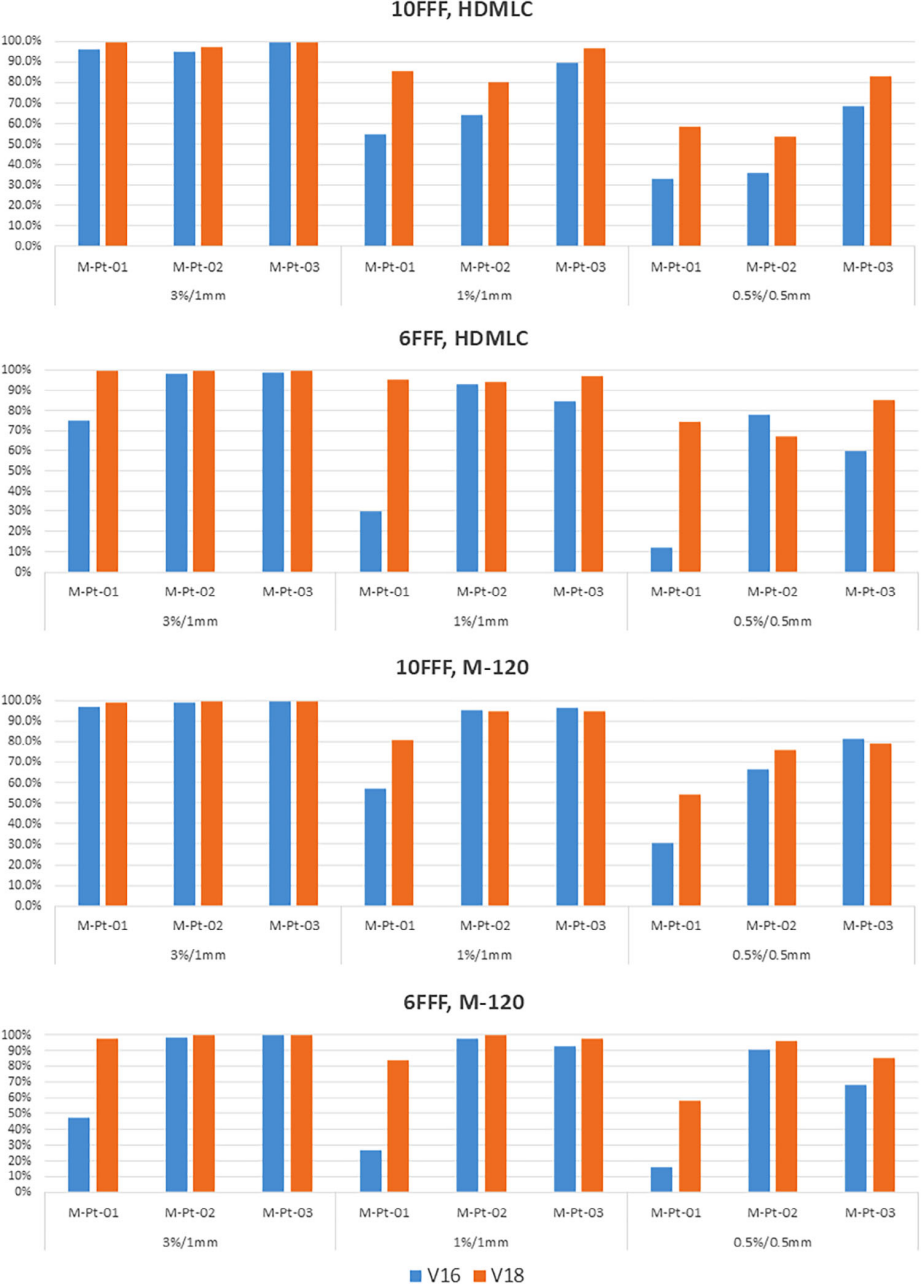
Gamma passing rate comparison at 3%/1 mm, 1%/1 mm, and 0.5%/0.5 mm between film measurements and calculations for the three SIMT patients. V16 is shown in blue and v18 in orange color.

**FIGURE 6:**
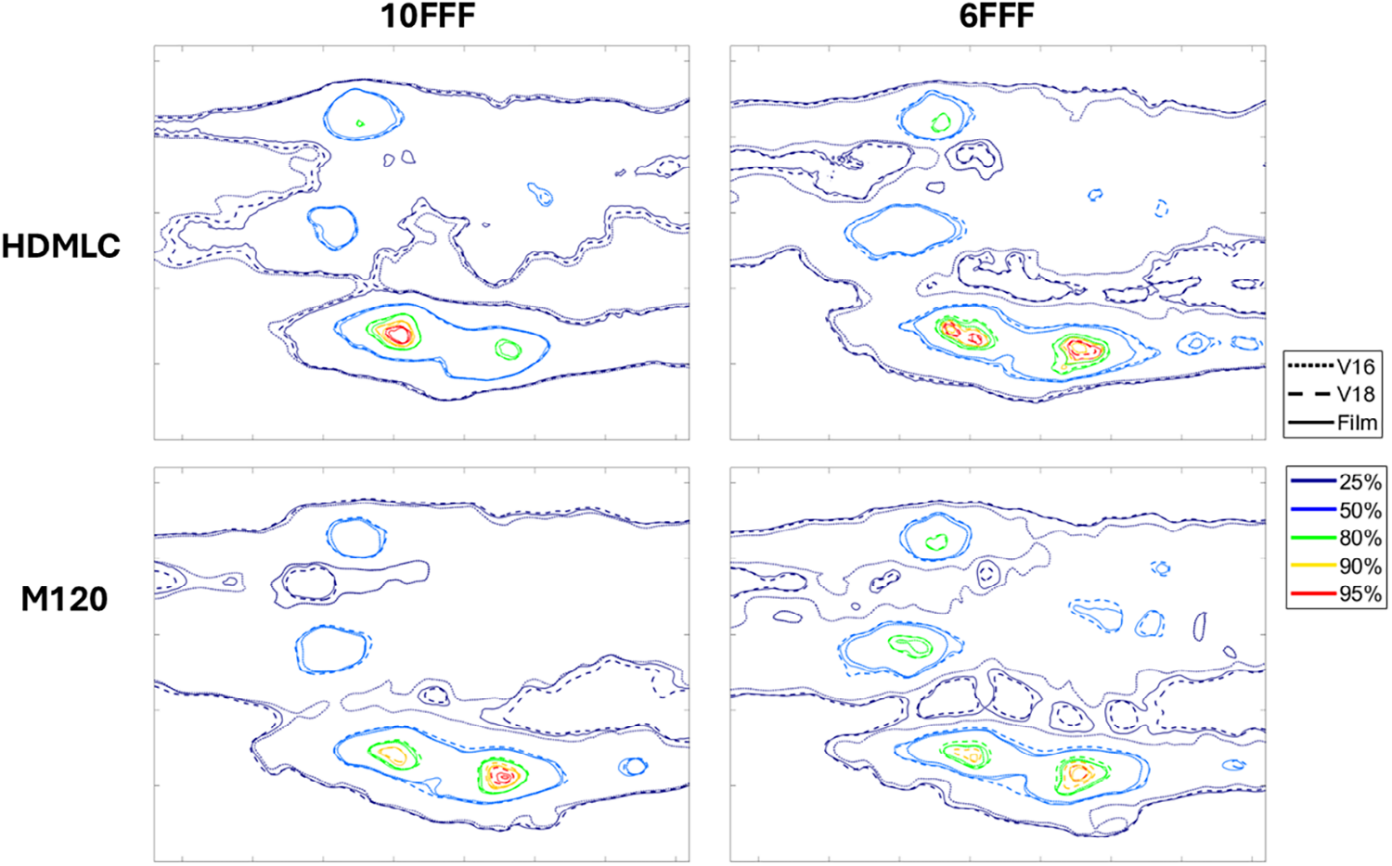
Isodose line comparison for patient M‐Pt‐01, showing film measurements (solid line), v16 model (dotted line), and v18 model (dashed line) at 25%, 50%, 80%, 90%, and 95% levels. All doses are normalized to the maximum dose of the v16 model.

For the SIMT patients, significant improvements in dose accuracy were observed using the v18 ELM model for both MLC types and energy levels. The gamma passing rate comparisons against film measurements are illustrated in Figure 5, while the isodose distribution comparisons for M‐Pt‐01 are presented in Figure 6.

In the complex case of M‐Pt‐01, which involved eight distributed small lesions, the v18 model greatly enhanced the dose agreement with film measurements for both HDMLC and M‐120 across 6FFF and 10FFF energies. Specifically, the 1%/1 mm passing rates increased by 31% for HDMLC‐10FFF, 65.6% for HDMLC‐6FFF, 23.9% for M‐120‐10FFF, and 57.6% for M‐120‐6FFF. Notably, the original v16 models exhibited passing rates of less than 30% for 6FFF, which improved to over 85% with the v18 model. The discrepancies of the v16 model are highlighted by the dotted isodose lines in Figure 6, which significantly deviate from the solid lines representing film measurements. For the 10FFF energy, the improvement with v18 is mainly observed in the low‐dose region, while the v18 model shows enhanced dosimetry in both low and high‐dose regions for the 6FFF energy.

The cases of M‐Pt‐02 and M‐Pt‐03 also demonstrated improvements with the v18 model for HDMLC‐10FFF, HDMLC‐6FFF, and M‐120‐6FFF. For M‐120‐10FFF, the v16 model achieved a passing rate improvement of less than 2% compared to v18. Similar results were observed using myQA SRS, as detailed in Table 5.

### 3.6 Dosimetrically equivalent machines

In this section, we aim to compare the differences in ELM parameters for matched machines. For the two matched TrueBeam machines, TB1 and TB2, the ELM parameters were measured and configured independently using the same setup and devices. Although both ELM models share the same transmission factor, there is a difference of 0.003 cm in DLG for both 10FFF and 6FFF.

**FIGURE 7:**
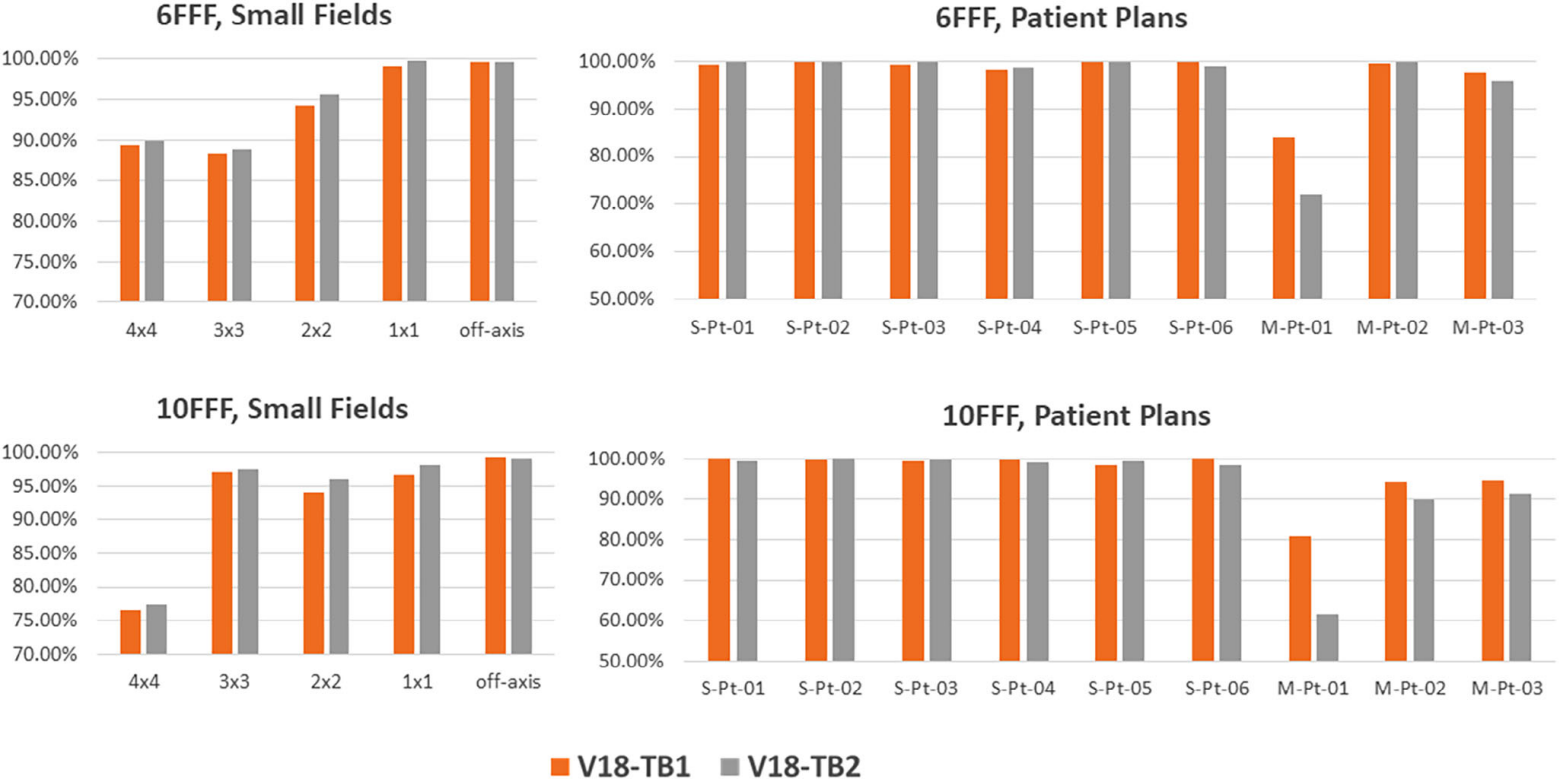
1%/1 mm gamma passing rate comparison against film measurements between TB1 and TB2, for small fields (left column) and patient plans (right column).

The two ELM models, v18‐TB1 and v18‐TB2, were evaluated against measurements performed on TB1 using films. The average gamma passing rate differences between v18‐TB1 and v18‐TB2 at 3%/2 mm, 3%/1 mm, and 1%/1 mm are summarized in Table 6. The comparison of the 1%/1 mm passing rate for each small field and patient plan is presented in Figure 7.

**TABLE 6:** Average gamma passing rate difference between v18‐TB1 and v18‐TB2 (v18‐TB1 ‐ v18‐TB2).

	3%/2 mm	3%/1 mm	1%/1 mm
6FFF small fields	0.0%	−0.2%	−0.6%
10FFF small fields	−0.1%	−0.5%	−0.9%
6FFF PSQA SIST	0.0%	−0.1%	−0.1%
10FFF PSQA SIST	0.0%	−0.1%	−0.2%
6FFF PSQA SIMT	1.1%	2.5%	4.6%
10FFF PSQA SIMT	1.4%	2.9%	9.1%

The measurements were performed on TB1 using films.

For small fields and SIST plans, the performance of the two models is quite similar. The v18‐TB2 model exhibits a slightly higher passing rate than v18‐TB1 in small fields, with a difference of less than 0.5% at 3%/1 mm, despite measurements being conducted on TB1. The main deviation between the two models is observed for SIMT patients, where v18‐TB1 demonstrates better agreement with the measurements. However, both ELM models show passing rates exceeding 99% at 3%/1 mm and 95% at 1%/1 mm, except for M‐Pt‐01, which presents challenges for the M‐120.

## 4 DISCUSSION

Accurate MLC modeling is essential for SRS, given the high levels of modulation and precision required in dose delivery. Traditional single parameter DLG models have frequently shown limitations in achieving the necessary accuracy, raising concerns about their reliability. Furthermore, the performance of the DLG model can be influenced by the user's experience, leading to extensive commissioning efforts. The introduction of the ELM model in the latest Eclipse version effectively addresses these challenges. By enhancing the accuracy of SRS dose calculations and streamlining the commissioning process, the ELM model not only facilitates more dependable dose delivery but also improves overall clinical efficiency.

Previous work^14^ has demonstrated the improved accuracy of the ELM model for both on‐axis and off‐axis sliding gap fields. This study extends this investigation by evaluating the accuracy of ELM for SRS compared to current clinical models. By assessing small fields and patient‐specific plans, including both on‐axis and distant off‐axis targets, we validate the effectiveness of ELM for SRS treatments. The ELM model was thoroughly evaluated across different MLC types, energies, and QA devices to provide comprehensive guidance for institutions planning to implement ELM in SRS treatments.

The measurement results indicate that the ELM model achieves similar or improved dose accuracy compared to the traditional binary DLG model. For on‐axis small fields, ELM shows enhanced agreement, particularly in the penumbra region, across different energies, MLC types, and measurement devices, with notable improvements for fields measuring 2 × 2 cm^2^ and smaller. In off‐axis small fields, the dose distribution is comparable between the ELM and DLG models in the direction perpendicular to leaf travel; along the leaf travel direction, ELM demonstrates superior alignment with measurements. The tongue‐and‐grove remain unchanged in ELM from the previous models.

When assessing patient plans, the ELM model reveals improvements in the 6FFF energy for both SIST and SIMT treatments, applicable to both HD‐MLC and M‐120 configurations, enhancing accuracy in both high and low‐dose regions. For 10FFF, while the performance of ELM for SIST with HD‐MLC remains consistent, an improvement is observed in SIMT cases. The M‐120 configuration shows gamma passing rates similar to those of the DLG model, yet ELM demonstrates better agreement at lower dose levels, particularly around the 20% and 50% thresholds. ELM proves especially effective for cases involving small, distributed targets, where the traditional DLG model often encounters significant challenges.

While the ELM model performs similarly to the original model for small fields and SIST with HDMLC 10FFF, it is important to note that the original DLG model for HDMLC 10FFF energy was iteratively fine‐tuned for SRS. The tuning process initially began with a DLG value validated for IMRT/VMAT treatments. In each iteration, the DLG value was adjusted, followed by recalculating SIST and SIMT patient plans and comparing them with measurements. This iterative process continued until satisfactory agreement between the DLG model and measurements was achieved. However, each iteration required several hours for recalculation and verification, and determining the truly optimal DLG remained elusive due to the limited time and effort that could be allocated to this tuning process. Consequently, while the fine‐tuned DLG model performed adequately for SIST patients, it still yielded unsatisfactory results for SIMT treatments.

In contrast, the ELM configuration eliminates the need for iterative leaf gap tuning. A one‐time configuration achieves excellent agreement with measurements. The overall dose accuracy of ELM is comparable to the fine‐tuned DLG model for SIST patients and shows substantial improvement for complex SIMT patients, significantly reducing the commissioning effort. For the models that were not originally adjusted for SRS, like HDMLC‐6FFF and M120, ELM provides a model inherently suited for SRS treatments. Therefore, for institutions with dedicated SRS beam models, ELM offers similar or even improved dosimetry. For institutions looking to establish an SRS program, ELM greatly enhances the efficiency and effectiveness of the commissioning process.

Regarding machine configurations, one set of ELM parameters provide similar dose agreement for small fields and SIST patients across dosimetrically equivalent machines. However, deviations arise in complex SIMT cases, where ELM configured on the delivering machine offers the highest accuracy. Therefore, using machine‐specific ELM parameters is more accurate in single‐machine settings. In cases where multiple machines are used and machine matching is required, a common set of ELM parameters can be established, similar to how DLG parameters were determined in Eclipse versions prior to v17. If high accuracy for SIMT SRS is a priority on a specific machine, it is worth carefully examining and validating the ELM parameters under SRS conditions and considering the creation of SRS‐specific models.

A recent study by Moktan et al. investigated the impact of the ELM on dose calculation accuracy for SIMT SRS treatments using HyperArc plans and concluded that the ELM improves target coverage and agreement with film measurements in high‐gradient regions.^15^ While both their work and ours focus on the clinical implementation of the ELM in SRS, there are notable differences in scope and methodology. Moktan et al. primarily evaluated the impact of ELM on dose accuracy for SIMT plans using the HDMLC and a limited number of targets and measurement points, with an emphasis on film‐based validation. In contrast, our study provides a broader evaluation across both HDMLC and Millennium 120 MLC systems, incorporates two photon energies (6FFF and 10FFF), and includes comparisons for small static fields, SIST, and SIMT plans. We also introduced independent validation using a high‐resolution CMOS detector array (myQA SRS) in addition to Gafchromic film. Furthermore, we examined inter‐machine consistency using two dosimetrically matched TrueBeam linacs and compared different ELM configurations. Thus, while our findings support the improvements in SRS modeling highlighted by Moktan et al., our study offers a comprehensive assessment of the ELM performance across MLC types, clinical scenarios, and measurement devices.

While Gafchromic films offer high spatial resolution and are widely used for small field dosimetry, they are subject to inherent limitations in absolute dose accuracy due to factors such as film uniformity, scanner variability, and dose‐response sensitivity. According to AAPM Task Group 235,^20^ the overall uncertainty in absolute film dosimetry is typically between 2% and 3% when careful calibration and scanning protocols are followed. In this study, we adhered to the recommendations outlined in TG‐235 for film calibration and scanning procedures. Nevertheless, some level of uncertainty remains inherent to film‐based measurements.

Gamma analysis criteria ranging from 3%/1 mm to 0.5%/0.5 mm were reported in the results. It is important to note that the 0.5%/0.5 mm criterion, while stringent, was primarily employed for relative comparison, rather than as an assessment of absolute dosimetric accuracy, and should be interpreted in the context of the measurement uncertainty.

For small field dosimetry, small‐volume water‐equivalent detectors such as plastic scintillation detectors or microDiamond detectors are recognized for their superior absolute dose accuracy. Unfortunately, such detectors were not available during the course of this study. To strengthen the validity of our measurements, all film‐based results were cross‐validated using a high‐resolution CMOS detector array (myQA SRS), providing an independent verification of spatial and dosimetric agreement.

While the overall agreement between Gafchromic film and myQA SRS measurements was strong, some isolated cases exhibited opposite trends in gamma passing rates between the two modalities. These differences are likely due to the distinct uncertainty profiles and operational characteristics of each measurement system. Gafchromic film provides high spatial resolution and is well‐suited for capturing steep dose gradients, but it is susceptible to absolute dosimetry uncertainties. In contrast, the myQA SRS system, based on a CMOS detector array, offers excellent reproducibility and submillimeter resolution but may be affected by limitations in detector density, angular dependence, and volume averaging effects—especially in low‐dose or off‐axis regions. A recent independent study^18^ found that discrepancies in gamma analysis using myQA SRS tend to occur when dose gradients align with detector spacing or when the device is rotated to capture complex off‐axis dose distributions, as was done in our SIMT measurements.

These findings highlight the importance of using complementary measurement tools for validation. While film provides superior spatial fidelity, myQA SRS adds robustness and efficiency in measurement. Together, they offer a more comprehensive assessment of the dose distribution and strengthen confidence in the observed trends, particularly in small field and stereotactic treatment settings.

## 5 CONCLUSION

The new ELM introduced in Eclipse v18 substantially improves efficiency and consistency of modeling process of the Eclipse dose calculation algorithm while maintaining comparable or superior accuracy for Linac‐based SRS. This advancement ensures more reliable dose delivery, particularly for complex SRS treatments, making it a valuable tool for institutions aiming to improve the accuracy and efficiency of their SRS programs.

## AUTHOR CONTRIBUTIONS

Conceptualization: TL, WG, MAB; Design: YY, TL, WG; Experiment: YY, KS, VL, MB, KT, WG; Analysis: YY, WG; Plan evaluation: MAB, SN; IRB: MAB, WG; Draft writing: YY, WG. All authors participated in contributing to text and the content of the manuscript, including revisions and edits. All authors approve of the content of the manuscript and agree to be held accountable of the work.

## CONFLICT OF INTEREST STATEMENT

The authors declare no conflicts of interest.
